# Prognostic value of haematological indices in sea turtles presenting for cold-stunning (sustained hypothermia)

**DOI:** 10.18849/ve.v8i1.561

**Published:** 2023-03-01

**Authors:** McCaide Wooten+

**Keywords:** SEA TURTLE, BLOOD, HAEMATOLOGY, BIOCHEMISTRY, COLD-STUNNING, COLD-STUN, ACIDOSIS

## Abstract

**PICO question:**

In sea turtles presenting for cold-stunning (sustained hypothermia), what blood analytes routinely evaluated at intake provide the most prognostic value?

**Clinical bottom line:**

**Category of research:**

Prognosis.

**Number and type of study designs reviewed:**

Ten studies were included in this evaluation including the following study designs: eight retrospective case series, one cross-sectional, and one retrospective cohort.

**Strength of evidence:**

Weak.

**Outcomes reported:**

The most consistent finding across all included studies in cold-stunned sea turtles was acidosis (suspected both respiratory or metabolic components) characterised by reduced blood pH, elevated partial pressure of carbon dioxide (pCO_2_), and reduced partial pressure of oxygen (pO_2_). However, this finding was not necessarily linked with failure of rehabilitation. Rather, sea turtles presenting for cold-stunning that did not survive rehabilitative therapy were typically in extreme states of homeostatic derangement involving acidosis, but often in conjunction with additional abnormalities (e.g. anaemia, sepsis, organ failure or dysfunction, pneumonia, etc.).

**Conclusion:**

As might be expected, the evaluated literature did not reveal a single or series of blood analytes that were definitively linked with the success or failure of rehabilitation in sea turtles presenting for cold-stunning. However, they did identify analytes that may provide the most clinical value in this clinical situation including packed cell volume (PCV), estimated white blood cell count (WBC), total and / or ionised calcium, pH, potassium (K), and lactate. Review of the available studies on the topic provides insightful information that can aid clinicians addressing this syndrome to triage and treat affected individuals most effectively. It also elucidated areas of opportunity for further research.

## How to apply this evidence in practice

The application of evidence into practice should take into account multiple factors, not limited to: individual clinical expertise, patient’s circumstances and owners’ values, country, location or clinic where you work, the individual case in front of you, the availability of therapies and resources.

Knowledge Summaries are a resource to help reinforce or inform decision making. They do not override the responsibility or judgement of the practitioner to do what is best for the animal in their care.

## The evidence

Based on the included studies, the most consistent biochemical finding in the blood of marine turtles presenting to rescue and rehabilitation studies for cold-stunning is acidosis, generally characterised by reduced blood pH, elevated partial pressure of carbon dioxide (pCO_2_), and reduced partial pressure of oxygen (pO_2_). At present, a respiratory or metabolic aetiology of this finding is undetermined, but it was suggested in several studies there are likely elements of both components contributing to the common finding given the nature of the syndrome.

It should be taken into consideration that nine of the ten studies (Innis et al., 2009; Powell et al., 2021; Stacy et al., 2013; Innis et al., 2007; Innis et al., 2019; Hunt et al., 2012; McNally & Innis, 2020; Keller et al., 2012; and Rockwell et al., 2017) evaluated here emanated from the same institution (The New England Aquarium, Boston, MA, USA), and as such are more subject to internal biases. However, this institution is also one of the most likely to be encountering cold-stunned sea turtles given its location along the northwest Atlantic, where juvenile and subadult animals with lower surface area-to-body mass ratios are found to frequent shallower coastal waters that are subject to the effects of rapid oceanic, meteorological, and atmospheric changes.

Also of note is the variability in study designs, outcomes of interest, and population sizes. These factors varied widely between the evaluated manuscripts and the strict evaluation of blood parameters as they pertained to the survival or death of cold-stunned turtles was infrequently the primary objective. Only three of seven known species of extant marine turtles were evaluated here, and of those, the Kemp’s ridley sea turtle (*Lepidochelys kempii*) is overrepresented, so blind generalisation of the findings herein is not advised; again, this is likely secondary to these reports primarily emanating from one institution and the nature of the Kemp’s ridley sea turtle being the most frequently encountered species succumbing to cold-stunning along the New England coastline. Further characterisation of blood parameters in cold-stunned sea turtles is needed in additional geographic regions and among additional species, where applicable, for better understanding of the physiological impacts of this syndrome. Given projections for increased cold-stunning events secondary to anthropogenically-influenced warming ocean surface temperatures (Griffin et al., 2019), opportunities for study and refinement of clinical methods for rehabilitation will only increase.

The overall presented evidence is deemed to be of ‘weak’ strength. However, the author finds the included materials to be of utmost academic, and particularly clinical, relevance.

## Summary of the evidence

**Innis et al. (2007):** 

Population:	Cold-stunned Kemp’s ridley sea turtles (*Lepidochelys kempii*) presenting to the New England Aquarium (USA) Rescue and Rehabilitation Department Oct 29–Nov 29, 2005.
Sample size:	26 sea turtles.
Intervention details:	On the day of admission and multiple times through ‘convalescence’, venous blood gas samples were collected aseptically with heparinised syringes and evaluated for pH, pCO_2_, pO_2_, bicarbonate (HCO_3_), plasma osmolality, sodium, potassium, chloride, ionised calcium, ionised magnesium, glucose, lactate, and blood urea nitrogen. ‘Convalescent’ turtles were those documented to be swimming in water at 25°C for at least 10 days, discontinued from parenteral fluid therapy for at least 7 days, and eating voluntarily daily for at least 7 days. 20 turtles were evaluated at each time point. 14 sea turtles provided data at both the initial and convalescent time points. Six turtles for which paired data were not available provided data at either the initial or convalescent time points. Applicable data were corrected to body temperature using species-specific and general correction methods as indicated.
Study design:	Retrospective case series.
Outcome studied:	Evaluation of differences between presenting and convalescent blood parameters in sea turtles surviving rehabilitation as compared to previously published data in both healthy and hypothermic turtles.
Main Findings (relevant to PICO question)	Blood samples were only analysed for turtles surviving rehabilitation. Samples from turtles that did not survive rehabilitation were not included in this study. The data produced under natural conditions were generally in agreement with blood values that had been experimentally derived in sea turtles exposed to a range of temperatures. There were consistent findings of hypermagnesemia, hypocalcaemia, and mild to moderate respiratory and metabolic acidosis among the turtles evaluated. Initial blood urea nitrogen (BUN) concentrations were significantly lower than convalescent concentrations. Initial lactate concentrations were higher than convalescent concentrations in the full data set, but not among paired individuals.
Limitations:	Small sample size. Strictly juvenile animals (however, inherently the most likely population given this syndrome, species, and geographic location). Single cold-stunning event period. Only surviving turtles were evaluated. Due to study timing, equipment availability, and animal transfers, only 14/26 turtles had both initial and convalescent samples collected. Different therapeutic interventions between individuals could potentially affect serial blood values.

**Innis et al. (2009):** 

Population:	Cold-stunned Kemp’s Ridley sea turtles (*Lepidochelys kempii*) presenting alive to the New England Aquarium (USA) between 2001–2005.
Sample size:	176 sea turtles.
Intervention details:	Blood samples were collected aseptically with un-heparinised syringes, transferred to heparinised blood collection tubes and submitted for evaluation by a commercial laboratory. Blood values were categorised as ‘initial’ values (within 5 days of hospitalisation), ‘clinically convalescent’ values, or ‘calculated convalescent’ values. Categorisation was dependent on the time of blood sample collection, the case outcome (survived vs died – if died, had to have died within 1 week of initial blood collection), and the clinical status of each turtle. ‘Clinically convalescent’ turtles (n = 15) were those that had no concurrent illness based on physical examination and radiographic findings, had completed treatments of fluids and antimicrobials at least 14 days prior to blood sample collection, had been in room temperatures of 70–80°F for at least 14 days prior to blood sample collection, and had eaten daily for at least 14 days prior to blood sample collection. ‘Calculated convalescent’ (n = 176) values were calculated using statistical analysis to define the time at which a variable stabilised during rehabilitation.
Study design:	Retrospective case series.
Outcome Studied:	Evaluated blood analytes included estimated white blood cell count (WBC), manual differential, haematocrit, alkaline phosphatase (ALP), alanine transaminase (ALT), aspartate aminotransferase (AST), creatine kinase (CK), lactate dehydrogenase (LDH), gamma-glutamyl transferase (GGT), albumin, total protein, globulin, bilirubin, blood urea nitrogen (BUN), creatinine, cholesterol, glucose, total calcium, phosphorus, chloride, potassium, sodium, and uric acid.
Main Findings (relevant to PICO question):	Initial total calcium, phosphorus, chloride, potassium, sodium, and uric acid concentrations were significantly greater in turtles that died (n = 34) than in turtles that survived (n = 142). Clinically convalescent values of BUN and total calcium were significantly greater than initial values among survivors. Clinically convalescent values of glucose, sodium, and uric acid were significantly less than initial values among survivors.
Limitations:	Strictly juvenile animals (however, inherently the most likely population given this syndrome, species, and geographic location). Only 15 turtles met the standards to be considered ‘clinically convalescent’. Different therapeutic interventions between individuals could potentially affect serial blood values.

**Anderson et al. (2011):** 

Population:	Cold-stunned green sea turtles (*Chelonia mydas*) retrieved from the shore or near-shore waters of eastern North Carolina (USA) Nov 2008, Feb 2009, and Dec 2009; control blood samples were collected from healthy green sea turtles caught in pound nets in conjunction with a tag-and-release study in Core Sound and Back Sound near Harker’s Island, North Carolina in Aug 2009, June 2010, and Sept 2010.
Sample size:	34 sea turtles.
Intervention details:	Blood samples were collected aseptically with un-heparinised syringes and immediately evaluated for venous pH, pCO_2_, pO_2_, bicarbonate, and ionised calcium; Packed cell volume (PCV) / Serum total solids (TS) were evaluated after collection; estimated total white blood cell count (WBC), manual differential counts, and a serum biochemistry panel were evaluated by a commercial laboratory. Cold-stunned turtles that were included in the study were those that: were recovered with similarly affected turtles following the passage of cold fronts during the periods of study, were comatose or near comatose, had no other evidence of trauma or disease, had a cloacal temperature <14°C, were live stranded, were examined by a veterinarian, and had blood samples collected prior to rewarming on the day of stranding. Applicable data were corrected to body temperature using species-specific and general correction methods as indicated.
Study design:	Cross-sectional study.
Outcome Studied:	Evaluation of differences in blood parameters between cold-stunned sea turtles (n = 22) at presentation and a healthy control population (n = 12).
Main Findings (relevant to PICO question):	All of the evaluated cold-stunned turtles survived to release except for one (21/22 [95.5%]). No significant differences in haematologic parameters were identified between the control and cold-stunned populations. Cold-stunned turtles had significantly lower total solids, serum total protein, albumin, glucose, total calcium, ionised calcium, sodium, chloride, and potassium values than control turtles. Cold-stunned turtles had significantly higher phosphorous, blood urea nitrogen, and uric acid values than control turtles. Cold-stunned turtles were mildly acidotic (uncharacterised as respiratory or metabolic).
Limitations:	Control population not sampled in the same season as sample population. Small sample size. Strictly juvenile animals (however, inherently the most likely population given this syndrome, species, and geographic location). All but one turtle survived, and the individual data for the deceased turtle is not explicitly stated. Different therapeutic interventions between individuals could potentially affect serial blood values.

**Hunt et al. (2012):** 

Population:	Cold-stunned Kemp’s ridley sea turtles (*Lepidochelys kempii*) admitted to the New England Aquarium (USA) Rescue and Rehabilitation Department. All sea turtles stranded Oct–Dec on the on the northern shore of Cape Cod from 2002–2008.
Sample size:	56 sea turtles, 87 plasma samples.
Intervention details:	Multiple times through rehabilitation, venous blood gas samples were collected aseptically with heparinised syringes for analysis. Collection days generally ranged within days 0–3 and 18–95 (i.e., ‘admission’ and ‘recovery’ samples, respectively). Samples were evaluated for corticosterone and free thyroxine using validated commercial assay kits. Glucose was recorded from either the same blood samples as the hormone assays or the nearest collected sample (majority within 7 days). White blood cell count (WBC) was assessed from separate samples attained within 2 days of admission or 7 days of recovery hormone samples. Activity and feeding behaviour were assessed daily. Turtles were characterised as ‘survivors’ (n = 31) if they lived until release or transfer to another institution. All others were considered ‘non-survivors’ (n = 25).
Study design:	Retrospective case series.
Outcome Studied:	Evaluation of corticosterone, free thyroxine, glucose, WBC, and activity / feeding behaviours in cold-stunned sea turtles and if there were any associations with survival.
Main findings (relevant to PICO question):	No significant differences were identified between corticosterone or free thyroxine levels of surviving and non-surviving turtles on admission. Corticosterone was significantly lower and free thyroxine was significantly higher in recovery samples of surviving turtles (not assessable in non-survivors). No differences in glucose were noted across groups. Corticosterone was negatively correlated with WBC in admission samples, but showed no correlation with WBC in recovery samples. WBC was significantly higher in survivors on admission than in either non-survivors on admission or in survivors during recovery. During recovery, free thyroxine was significantly and positively correlated with grams of food ingested per day during the week prior to sampling. Turtles scored as ‘active’ during the week prior to sampling had significantly higher free thyroxine than turtles scored as ‘quiet’.
Limitations:	Corticosterone samples were very likely influenced by handling and transport necessitated during admission. No control group. Different therapeutic interventions between individuals could potentially affect serial blood values. Strictly juvenile animals (however, inherently the most likely population given this syndrome, species, and geographic location). Poor availability of normal data for corticosterone and free thyroxine levels in free-ranging healthy sea turtles. Inconsistent sampling regimen (influenced by retrospective nature).

**Keller et al. (2012):** 

Population:	Cold-stunned Kemp’s ridley sea turtles (*Lepidochelys kempii*) admitted to the New England Aquarium (USA) from Oct 2005 to Nov 2009.
Sample size:	64 sea turtles (32 matched pairs).
Intervention details:	Turtles were grouped by survivorship with ‘non-survivors’ characterised as dying of natural causes within the first 3 days of hospitalisation (n = 32). ‘Survivors’ were paired with each ‘non-survivor’ based on time of hospitalisation (within 2 days) (n = 32). Blood was collected by heparinised syringe and evaluated on day 1 of hospitalisation and day of death for ‘non-survivors’ and corresponding hospitalisation days for ‘survivors’. Applicable data were corrected to body temperature using species-specific and general correction methods as indicated.
Study design:	Retrospective case series (defined in manuscript as case-control, but controls were a subset of identified cases).
Outcome studied:	Variables of interest included blood pH, pCO_2_, pO_2_, haematocrit, anion gap, osmolality, ionised calcium, ionised magnesium, sodium, potassium, chloride, glucose, lactate, bicarbonate, and blood urea nitrogen concentrations.
Main findings (relevant to PICO question):	pO_2_, pH, and bicarbonate were significantly lower on day 1 in non-survivors than in survivors. pCO_2_ and potassium concentrations were significantly greater on day 1 in non-survivors than in survivors. Compared with survivors, there were significantly greater percentage changes for ionised calcium, potassium, and lactate concentrations in non-survivors. Compared with survivors, there were significantly lower percentage changes for pH, glucose, and bicarbonate concentrations in non-survivors.
Limitations:	Strictly juvenile animals (however, inherently the most likely population given this syndrome, species, and geographic location). Different therapeutic interventions between individuals could potentially affect serial blood values. ‘Controls’ are defined as a subset of the population of study rather than being an unaffected population.

**Stacy et al. (2013):** 

Population:	Cold-stunned Kemp’s ridley sea turtles (*Lepidochelys kempii*) that were admitted to the New England Aquarium (USA) between Oct 2010 and Dec 2011 and possessed a complete set of blood analytes at admission. Turtles that died had to have died within 7 days of admission (n = 25).
Sample size:	143 sea turtles.
Intervention details:	Blood was collected by heparinised syringe and evaluated on admission prior to any treatments. Applicable data were corrected to body temperature using species-specific and general correction methods as indicated. Three mortality prediction indices (MPI1, MPI2, MPI3) were created to objectively evaluate and quantify the severity of observed biochemical derangements. Within the three indices, analytes with receiver operating characteristic (ROC) curve area under the curve (AUC) values greater than or equal to 0.7 were included in development of three final mortality prediction indices (MPI4, MPI5, MPI6).
Study design:	Retrospective case series.
Outcome studied:	Variables of interest included blood pH, pCO_2_, and pO_2_; concentrations of sodium, potassium, chloride, ionised calcium, and glucose; osmolality, and anion gap.
Main findings (relevant to PICO question):	The final three MPIs were not statistically different from one another in predicting mortality with the highest sensitivity and specificity combination (MPI6) reported at 88% and 80.51%, respectively. Analytes included in the final MPIs were pH, pCO_2_, pO_2_, and potassium (pH and potassium were included in all three). In conjunction with other clinical factors, the developed MPIs could aid clinicians in making evidence-based decisions for cold-stunned sea turtles.
Limitations:	Strictly juvenile animals (however, inherently the most likely population given this syndrome, species, and geographic location). Mortality rate excludes mortalities that occurred prior to admission. Limited geographic range of studied population.

**Rockwell et al. (2017):** 

Population:	Cold-stunned Kemp’s ridley sea turtles (*Lepidochelys kempii*) that stranded on Massachusetts (USA) beaches in 2012 and 2013.
Sample size:	202 sea turtles.
Intervention details:	Turtles were included that were found alive and were transported for hospitalisation, as well as turtles that were found alive but died prior to transport. Blood was collected by heparinised syringe and evaluated on intake. Turtles were categorised as an ‘immediate intake’ if the date of their stranding record (date discovered on the beach) and date of their intake physical exam (date admitted to the hospital) matched (n = 120). If the date of the intake physical exam was on the day after their stranding record, they were categorised as a ‘delayed intake’ (n = 82). Outcome results were categorised as: dead on arrival (DOA) (n = 22), mortality by natural causes while in care (n = 20), mortality by euthanasia while in care (n = 6), and released (n = 154). Turtles were also grouped as released or not released, with not released containing all those that were DOA, died naturally, or were euthanised. Applicable data were corrected to body temperature using species-specific and general correction methods as indicated.
Study design:	Retrospective cohort study.
Outcome studied:	Overall effect of hospitalisation timing on cold-stunned Kemp’s ridley sea turtles. Evaluated blood parameters were packed cell volume (PCV), total solids, sodium, potassium, chloride, ionised calcium, ionised magnesium, glucose, lactate, blood urea nitrogen (BUN), haematocrit, pCO_2_, pO_2_, and pH.
Main findings (relevant to PICO question):	Data were analysed from all 202 turtles, regardless of rehabilitative success. PCV, total solids, and potassium concentration were significantly lower in sea turtles that had an immediate intake compared to those that had a delayed intake. There was no significant difference in outcome (released vs. not released) based on intake time. There was no significant difference in any individual outcome between turtles with immediate intake versus turtles with delayed intake.
Limitations:	Potential selection bias for immediate vs delayed hospitalisation (i.e., subjectively ‘worse’ cases immediately presented to hospital earlier), although no statistical differences were noted. Strictly juvenile animals (however, inherently the most likely population given this syndrome, species, and geographic location). Limited geographic range of studied population. Different therapeutic interventions between individuals could potentially affect serial blood values.

**Innis et al. (2019):** 

Population:	Cold-stunned loggerhead sea turtles (*Caretta caretta*) admitted to New England Aquarium (USA) after stranding on Cape Cod, Massachusetts (USA) between 2008 and 2016.
Sample size:	155 sea turtles.
Intervention details:	Turtles with blood data from the first day of hospitalisation were sorted into ‘non-survivor’ (death within first week of hospitalisation) (n = 20) and ‘survivor’ (survived to be released into the wild) (n = 135) groups. Blood was collected by heparinised syringe and evaluated on intake for all turtles and at least once more for all ‘survivor’ turtles (the most recent blood data for ‘survivor’ turtles was utilised as the convalescent sample). Applicable data were corrected to body temperature using Kemp’s ridley sea turtle (*Lepidochelys *kempii) species-specific and general correction methods as indicated.
Study design:	Retrospective case series.
Outcome studied:	Evaluated blood analytes included blood urea nitrogen, ionised calcium, ionised magnesium, chloride, creatinine, glucose, potassium, lactate, sodium, pCO_2_, pO_2_, pH, and bicarbonate.
Main findings (relevant to PICO question):	Data were analysed from all 155 turtles, regardless of rehabilitative success. Non-survivors had significantly higher glucose, potassium, lactate, and pCO_2_ than survivors at admission. Non-survivors had significantly lower pH and pO_2_ than survivors at admission. Comparison of initial vs convalescent data for survivors indicated that convalescent turtles had significantly higher blood urea nitrogen (BUN), sodium, chloride, pH, and ionised calcium, as well as significantly lower glucose, lactate, and bicarbonate.
Limitations:	The inclusion of data acquired using two different blood analyser units was presumed, though not confirmed, to result in significant differences between 4/13 blood parameters (noted by authors to be of minimal clinical significance). Variation in timing of acquisition of convalescent samples. Limited geographic range of studied population. Use of non-species-specific temperature correction methods (although another marine chelonian species). Different therapeutic interventions between individuals could potentially affect serial blood values.

**McNally & Innis (2020):** 

Population:	Cold-stunned, primarily subadult, loggerhead sea turtles (*Caretta caretta*) admitted to New England Aquarium (USA) after stranding on Cape Cod, Massachusetts (USA) between 2008 and 2016.
Sample size:	133 sea turtles.
Intervention details:	Turtles with blood data from the within the first week of hospitalisation were sorted into ‘initial died’ (first sample, death while hospitalised) (n = 4), ‘initial survived’ (first sample, survived to be released into the wild) (n = 129), and convalescent (last convalescent sample prior to release if released from New England Aquarium) (n = 24). Blood was collected by heparinised syringe and evaluated by a commercial laboratory.
Study design:	Retrospective case series.
Outcome studied:	Evaluated blood parameters included estimated white blood cell count (WBC), manual differential, haematocrit, alkaline phosphatase (ALP), alanine transaminase (ALT), aspartate aminotransferase (AST), creatine kinase (CK), lactate dehydrogenase (LDH), gamma-glutamyl transferase (GGT), albumin, total protein, globulin, bilirubin, blood urea nitrogen (BUN), creatinine, cholesterol, glucose, total calcium, phosphorus, chloride, potassium, sodium, and uric acid.
Main findings (relevant to PICO question):	Convalescent WBC count and absolute and relative heterophil counts were significantly lower than initial values, whereas absolute and relative eosinophil counts were higher. Convalescent values for CK, LDH, glucose, and uric acid were significantly lower than initial values. Convalescent ALT, AST, albumin, GGT, total protein, globulin, BUN, total calcium, phosphorus, chloride, and potassium were significantly higher than initial values.
Limitations:	Convalescent results only available from 24 turtles. Statistical comparison of turtles that died and turtles that survived could not be completed due to limited sample size. Initial data not collected until day 3 or 4 of hospitalisation, likely skewing results. Different therapeutic interventions between individuals could potentially affect serial blood values.

**Powell et al. (2021):** 

Population:	Cold-stunned Kemp’s ridley sea turtles (*Lepidochelys kempii*) that stranded on Massachusetts (USA) beaches between 2008 and 2018 and were identified from historical medical records by use of the search terms: osteomyelitis, osteolysis, osteolytic, osteotomy, arthritis, remodeling, lysis, humerus, humeral, tarsus, tarsal, carpus, carpal, tibia, ulna, radius, scapula, femur, *Enterococcus*, *Mycobacterium*, and *Serratia*.
Sample size:	25 sea turtles.
Intervention details:	Blood was collected and sorted according to timing of radiographic diagnosis of osteolytic lesions (‘initial’ median of 10 days prior to diagnosis, ‘convalescent’ of 22 days after cessation of antimicrobial therapy) evaluated by a commercial laboratory.
Study design:	Retrospective case series.
Outcome studied:	Characterise osteolytic lesions in cold-stunned Kemp’s ridley sea turtles hospitalised for rehabilitation and describe methods used for the management of such lesions. Evaluated blood parameters included estimated white blood cell count (WBC), manual differential, haematocrit, alkaline phosphatase (ALP), alanine transaminase (ALT), aspartate aminotransferase (AST), creatine kinase (CK), lactate dehydrogenase (LDH), gamma-glutamyl transferase (GGT), albumin, total protein, plasma protein, globulin, albumin:globulin ratio, bilirubin, blood urea nitrogen (BUN), creatinine (Cr), BUN:Cr ratio, cholesterol, glucose, total calcium, phosphorus, chloride, potassium (K), sodium, Na:K ratio, total CO_2_, anion gap, and uric acid.
Main findings (relevant to PICO question):	Compared with convalescent values, haematologic and biochemical data at the time of radiographic diagnosis were significantly higher for WBC, absolute heterophil, absolute and relative monocyte, absolute and relative basophil, and relative eosinophil counts; plasma chloride and cholesterol concentrations; and alkaline phosphatase activity. Plasma albumin, total protein, phosphorus, and potassium concentrations were significantly lower at the time of radiographic diagnosis.
Limitations:	Small sample size, initial and convalescent data only available for 21 individuals. The primary objective of this study was not to evaluate blood analyte differences between turtles over time, but rather to evaluate osteomyelitis in cold-stunned turtles. Potential for skewing of blood data secondary to all affected individuals having some degree of osteomyelitis. Blood was evaluated associated with timing of osteolytic lesion diagnosis rather than time of hospital admission / release. Different therapeutic interventions between individuals could potentially affect serial blood values.

## Appraisal, application and reflection

For clarity, the most relevant haematological and biochemical parameters evaluated in the included studies are categorised, analysed, and summarised below:

### Haematological indices

Elevated packed cell volume* (PCV) values at first blood evaluation of affected turtles were observed in multiple studies. In general, this finding was attributed to haemoconcentration given the tendency for convalescent PCV values to decrease in conjunction with other biochemical indicators of dehydration (Innis et al., 2009; Keller et al., 2012; and Rockwell et al., 2017). Keller et al. (2012) and Innis et al. (2009) also indicate a wide range of presenting PCVs, with several individuals presenting with anaemia. This finding tended to be associated with individuals that presented with one or more chronic comorbidities and was not necessarily found to be directly associated with cold-stunning.^1^

Regarding the leukogram, a common finding was for cold-stunned animals to present with elevated total white blood cell counts (WBC) (Innis et al., 2009; Powell et al., 2021; Hunt et al., 2012; and McNally & Innis, 2020). McNally and Innis (2020) postulated the elevated WBC observed in their study to most likely be linked with stress given the concurrent findings of heterophilia, lymphopenia, eosinopenia, and increased heterophil-to-lymphocyte ratio. As Hunt et al. (2012) showed, a component of corticosterone influence appears likely in cold-stunning cases. Interestingly, that study also revealed that non-surviving turtles had significantly lower WBCs at admission compared to survivors; although the finding of total leukopenia was likely multi-factorial in origin, this finding may suggest that leukopenia in the context of cold-stunning could be a negative prognostic indicator.

*It should also be acknowledged that the evaluated manuscripts use both ‘packed cell volume’ and ‘haematocrit’ to describe the metric derived by determining the percent volumetric contribution of red blood cells to a whole blood sample following centrifugation. By definition, this is a ‘packed cell volume’. ‘Haematocrit’ is the terminology given to the metric generated by multiplying mean cell volume and red blood cell count that have been directly measured by an automated haematology analyser. However, since there are currently no haematology analysers calibrated to evaluate avian or herptile blood samples due to the nucleation of red blood cells in these species, it is not necessarily incorrect to use these terms interchangeably in this context.

### Potassium

Significantly higher potassium (K) concentrations were reported in non-surviving turtles vs surviving turtles on initial evaluation in three different studies (Innis et al., 2009; Innis et al., 2019; and Keller et al., 2012). K values were also included in all mortality prediction indices (MPIs) developed by Stacy et al. (2013) with higher values associated with higher index values. All of these studies evaluated potassium in Kemp’s ridley turtles, and the corresponding findings were consistent with previously reported potassium findings in cold-stunned sea turtles (Carminati et al., 1994; and Turnbull et al., 2000). Conversely, several studies found initially decreased K levels relative to a control population (Anderson et al., 2011) and convalescent values in recovered turtles (Powell et al., 2021; and McNally & Innis, 2020). These three studies evaluated green (Anderson et al., 2011) and loggerhead (Powell et al., 2021; and McNally & Innis, 2020) turtles.

The observed discord between studies regarding potassium is not fully explained. The observation by Anderson et al. (2011) that differences in potassium concentrations ‘may reflect differences in species, environmental conditions, sampling times, and methodology among studies’ (p. 252) remains poignant given the currently available evidence; animal age (juvenile vs subadult), chronicity of hypothermia, renal functionality, and blood pH status (Lutz et al., 1989) are other suggested factors at play. However, what does appear to be clear is that there is a negative prognostic relationship with severe hyperkalaemia in the Kemp’s ridley.

### Acid-base indices

Contrary to what is reported in experimental studies of blood pH and hypothermia in sea turtles (Kraus et al., 1980; Lutz et al., 1989; and Moon et al., 1997), the presence of relative physiological acidosis in cold-stunned turtles was noted across multiple papers (with temperature correction) (Anderson et al., 2011; Keller et al., 2012; Innis et al., 2007; Innis et al., 2019; Rockwell et al., 2017; and Stacy et al., 2013).Metabolic and respiratory origins for this finding are both supported due to hypoventilation (with secondary hypercarbia and anaerobic metabolic processes), reduced perfusion at lower body temperatures, reduced / exhausted bicarbonate buffering capacity, and reduced renal clearance. Keller et al. (2012) reported significantly increased pCO_2_ and significantly reduced pO_2_, pH, and HCO_3_ in non-surviving turtles compared to those that survived cold-stunning. Mortalities in the Innis et al. (2019) report were also linked with significantly elevated pCO_2_ and significantly reduced pH and pO_2_ relative to surviving loggerheads. pH, pCO_2_, and pO_2_ were also included across all MPIs developed by Stacy et al. (2013) where decreased pH, elevated pCO_2_, and decreased pO_2_ increased likelihood of mortality. This evidence supports the use of marked blood gas derangements, when corrected for temperature, for inclusion as a negative prognostic indicator in cold-stunned turtles.

### Calcium

Relative to convalescent values, initial ionised calcium (Innis et al., 2007; Innis et al., 2019; and Keller et al., 2012) or total calcium (Innis et al., 2009; and McNally & Innis, 2020) values of surviving turtles were reduced at presentation across multiple studies. Anderson et al. (2011) also noted hypocalcaemia (total and ionised) in cold-stunned individuals relative to controls. Interestingly, for turtles that did not survive rehabilitation, Keller et al. (2012) reported significant increases in ionised calcium over the first few days of hospitalisation relative to survivors; Innis et al. (2007) also showed significantly increased total calcium among non-survivors at presentation relative to survivors. The mechanism for this disparity has not been fully described, but for the turtles that died, the authors suggested that compromised renal function, loss of cation homeostatic mechanisms, compensatory homeostatic responses to acidosis, and / or iatrogenic causes could be factoring into the observation. Given these observations, the prognostic value of ionised or total calcium are still undetermined. Hypocalcaemia appears that it should be an expected finding in cold-stunned turtles, but further investigation into calcium status as it pertains to this syndrome is needed to describe how changes in calcium over time might be associated with mortality.

### Lactate

The use of point- and serial-lactate evaluation as a means of identifying the magnitude of anaerobic metabolism occurring in domestic dogs and cats presenting to emergency facilities has become a classic example of a prognostic test in the veterinary literature in recent years (Kohen et al., 2018; Saint-Pierre et al., 2021; and Zacher et al., 2010). In the evaluated studies, lactate values in cold-stunned turtles did not exceed levels reported in healthy sea turtles exposed to forced submergence, extended voluntary dives, or trawl net capture (Berkson, 1966; Lutz et al., 1985; Stabenau et al., 1991; and Wood et al., 1984). Innis et al. (2007) noted that initial lactate concentrations were significantly elevated relative to convalescent values. A later study showed significantly greater lactate levels in turtles that died vs those that survived (Innis et al., 2019). Keller et al. (2012) demonstrated evidence of significantly different net positive change of lactate values (despite medical intervention) over the first 2 to 3 days of hospitalisation in cold-stunned turtles that died relative to net negative change in turtles that survived over the same period. This latter study showed that there may be a negative prognostic relationship with serially increasing lactate concentrations following hospitalisation for cold-stunning. More recent evidence has shown that serum lactate levels in clinically healthy loggerhead turtles restrained for health exams increase significantly over 15 minutes of restraint (Mones et al., 2021). In this context, a clinician must keep in mind the time at which a lactate sample, or series of samples, was taken in order to appropriately interpret the findings.

### Free thyroxine

Though only evaluated in one of the included manuscripts (Hunt et al., 2012) and not necessarily identified as a prognostic indicator for release, free thyroxine (fT4) is identified here given its potential usefulness in connection to return to normal function in sea turtles being rehabilitated for cold-stunning. Hunt et al. (2012) showed that in the week prior to sampling, hospitalised turtles that were documented to be eating every day and scored as ‘active’ (vs ‘quiet’) had significantly increased fT4 concentrations over those that were not. This finding shows value for further investigation into thyroid hormone activity in sea turtles and the potential application of T4 supplementation in sea turtles being rehabilitated for cold-stunning.

### Conclusions

The summative findings of the reviewed articles most strongly indicate the primary pathological effect of cold-stunning in the studied species of sea turtles, particularly the Kemp’s ridley, to be a physiological acidosis (likely both metabolic and respiratory in origin). Secondary imbalances in electrolyte status, immune status, and cation balance appear to ensue in a compensatory fashion and anaerobic metabolic processes generally coincide. Frequently, sea turtles presenting for cold-stunning may also present with other comorbidities (sepsis, pneumonia, anaemia, etc.). Prognostically, total leukopenia, extreme derangements of blood acid-base parameters (increased pCO_2_, decreased pO_2_, reduced blood pH), severe hyperkalaemia, serially increasing blood lactate concentrations, and possibly rapid increases in total or ionised calcium have been identified in association with increased likelihood of death.

Areas identified for potential research include, but are not limited to, further characterisation of the metabolic processes affected by the speed of rewarming and optimisation to prevent exacerbation of the acidotic state in cold-stunned turtles, description of baseline thyroid activity in healthy sea turtles, implementation of thyroid supplementation in turtles showing reduced activity and / or hyporexia while in rehabilitation, evaluation of serial lactate values across sea turtle species presenting for rehabilitation, and comparative study of blood, gross pathological, and histopathological findings in sea turtles that succumb to cold-stunning.

The current recommendations for rehabilitation of cold-stunned sea turtles (Innis & Staggs, 2017; and Wyneken et al., 2006) generally result in favourable outcomes with 50–80% of animals being successfully rehabilitated and released (Innis & Staggs, 2017). When turtles that succumb to the syndrome within the first 3 days of treatment are excluded, that statistic approaches 90% (Innis & Staggs, 2017; and Wyneken et al., 2006). With continued improvement of the existent protocols designed to remedy this syndrome for the vulnerable species experiencing it, these statistics can only improve.

## Methodology

**Search Strategy:** 

Databases searched and dates covered:	CAB Abstracts via CAB Direct, 1973–Present PubMed via National Library of Medicine, 1902–Present Web of Science Zoological Record via Clarivate Analytics, 1864–Present
Search strategy:	CAB Abstracts: 1) sea turtle* OR marine turtle* OR cheloni* 2) cold-stun* OR hypothermia 3) biomarker* OR blood* OR hemat* OR chemistr* OR biochem* OR analyte* OR plasma OR ser* 4) 1 AND 2 AND 3 PubMed: ((sea turtle* OR marine turtle* OR cheloni*) AND (cold-stun* OR hypothermia)) AND (biomarker* OR blood* OR hemat* OR chemistr* OR biochem* OR analyte* OR plasma OR ser*) Web of Science Zoological Record: ((TS=(sea turtle* OR marine turtle* OR cheloni*)) AND TS=(cold-stun* OR hypothermia)) AND TS=(biomarker* OR blood* OR hemat* OR chemistr* OR biochem* OR analyte* OR plasma OR ser*) Web of Science Zoological Record: ((TS=(sea turtle* OR marine turtle* OR cheloni*)) AND TS=(cold-stun* OR hypothermia)) AND TS=(biomarker* OR blood* OR hemat* OR chemistr* OR biochem* OR analyte* OR plasma OR ser*)
Dates searches performed:	06 Dec 2022

**Exclusion / Inclusion Criteria:** 

Exclusion:	Non-English-language manuscripts, case reports, abstracts, reviews, book chapters, other non-journal articles, repeat of same relevant result within search.
Inclusion:	Relevant journal articles, must include evaluation blood analytes in cold-stunned marine turtle species.

**Search Outcome:** 

Database	Number of results	Excluded – Does not include blood analyte evaluation	Excluded – Blood analyte data left unpublished	Excluded – Blood analyte data left unpublished	Excluded – Evaluates non-biological blood analyte(s)	Excluded – Blood analytes are not routinely evaluated	Excluded – Non-journal article	**Total relevant papers**
CAB Abstracts	27	11	1	4	1	1	1	8
PubMed	15	4	1	3	0	1	0	6
Web of Science	28	10	1	3	1	2	1	10
Total relevant papers when duplicates removed								10

## ORCID

McCaide Wooten: https://orcid.org/0000-0001-8121-0824

## Conflict of Interest

The author declares no conflicts of interest.

## References

[BIBR-1] Anderson E.T., Harms C.A., Stringer E.M., Cluse W.M. (2011). Evaluation of hematology and serum biochemistry of cold-stunned green sea turtles (Chelonia mydas) in North Carolina, USA. Journal of Zoo and Wildlife Medicine.

[BIBR-2] Berkson H. (1966). Physiological adjustments to prolonged diving in the Pacific green turtle (Chelonia mydas agassizii. Comparative Biochemistry and Physiology.

[BIBR-3] Carminati C., E Gerle, LL. Kiehn, RP Pisciotta (1994). Blood chemistry comparison of healthy vs. hypothermic juvenile Kemp’s ridley sea turtles (Lepidochelys kempii) in the New York Bight. Proceedings of the 14th Annual Workshop on Sea Turtle Conservation and Biology.

[BIBR-4] Griffin L.P., Griffin C.R., Finn J.T., Prescott R.L., Faherty M., Still B.M., Danylchuk A.J. (2019). Warming seas increase cold-stunning events for Kemp’s ridley sea turtles in the northwest Atlantic. PLoS ONE.

[BIBR-5] Hunt K.E., Innis C., Rolland R.M. (2012). Corticosterone and thyroxine in cold-stunned Kemp’s ridley sea turtles (Lepidochelys kempii. Journal of Zoo and Wildlife Medicine.

[BIBR-6] Innis C.J., Tlusty M., Merigo C., Weber E.S. (2007). Metabolic and respiratory status of cold-stunned Kemp’s ridley sea turtles (Lepidochelys kempii. Journal of Comparative Physiology. B, Biochemical, Systemic, and Environmental Physiology.

[BIBR-7] Innis C.J., Ravich J.B., Tlusty M.F., Hoge M.S., Wunn D.S., Boerner-Neville L.B., Merigo C., Weber E.S. (2009). Hematologic and plasma biochemical findings in cold-stunned Kemp’s ridley turtles: 176 cases (2001–2005. Journal of the American Veterinary Medical Association.

[BIBR-8] Innis C., Staggs L., Manire C., Norton T., Stacy B., Innis C., Harms C. (2017). Cold-Stunning. Sea Turtle Health & Rehabilitation.

[BIBR-9] Innis C.J., Divers S., Stahl S. (2019). Medical management and rehabilitation of sea turtles. Mader’s Reptile and Amphibian Medicine and Surgery.

[BIBR-10] Innis C.J., McGowan J.P., Burgess E.A. (2019). Cold-stunned loggerhead sea turtles (Caretta caretta): initial vs. convalescent physiologic status and physiologic findings sssociated with death. Journal of Herpetological Medicine and Surgery.

[BIBR-11] Keller K.A., Innis C.J., Tlusty M.F., Kennedy A.E., Bean S.B., Cavin J.M., Merigo C. (2012). Metabolic and respiratory derangements associated with death in cold-stunned Kemp’s ridley turtles (Lepidochelys kempii): 32 cases (2005–2009. Journal of the American Veterinary Medical Association.

[BIBR-12] Kohen C.J., Hopper K., Kass P.H., Epstein S.E. (2018). Retrospective evaluation of the prognostic utility of plasma lactate concentration, base deficit, pH, and anion gap in canine and feline emergency patients. Journal of Veterinary Emergency and Critical Care.

[BIBR-13] Kraus D.R., Jackson D.C. (1980). Temperature effects on ventilation and acid-base balance of the green turtle. American Journal of Physiology-Regulatory, Integrative and Comparative Physiology.

[BIBR-14] Lutz P.L., Bentley T.B. (1985). Respiratory Physiology of Diving in the Sea Turtle. Copeia.

[BIBR-15] Lutz P.L., Bergey A., Bergey M. (1989). Effects of temperature on gas exchange and acid-base balance in the sea turtle Caretta caretta at rest and during routine activity. Journal of Experimental Biology.

[BIBR-16] McNally K.L., Innis C.J. (2020). Plasma biochemistry and hematologic values of cold-stunned loggerhead sea turtles (Caretta caretta. Journal of Herpetological Medicine and Surgery.

[BIBR-17] Mones A.B., Gruber E.J., Harms C.A., Lohmann C.M.F., Lohmann K.J., Lewbart G.A. (2021). Lactic acidosis induced by manual restraint for health evaluation and comparison of two point-of-care analyzers in healthy loggerhead sea turtles (Caretta caretta. Journal of Zoo and Wildlife Medicine.

[BIBR-18] Moon D.Y., MacKenzie D.S., Owens D.W. (1997). Simulated hibernation of sea turtles in the laboratory: I. feeding, breathing frequency, blood pH, and blood gases. The Journal of Experimental Zoology.

[BIBR-19] Powell A.L., Tuxbury K.A., Cavin J.M., Stacy B.A., Frasca S., Stacy N.I., Brisson J.O., Solano M., Williams S.R., McCarthy R.J., Innis C.J. (2021). Osteomyelitis in cold-stunned Kemp’s ridley sea turtles (Lepidochelys kempii) hospitalized for rehabilitation: 25 cases (2008–2018. Journal of the American Veterinary Medical Association.

[BIBR-20] Rockwell K.E., Innis C.J., Merigo C., Prescott R. (2017). The effect of delayed hospitalization in cold-stunned Kemp’s ridley turtles (Lepidochelys kempii. Journal of Herpetological Medicine and Surgery.

[BIBR-21] Saint-Pierre L.M., Hopper K., Epstein S.E. (2021). Retrospective evaluation of the prognostic utility of plasma lactate concentration and serial lactate measurements in dogs and cats presented to the emergency room (January 2012 – December 2016): 4863 cases. Journal of Veterinary Emergency and Critical Care.

[BIBR-22] Stabenau E.K., Heming T.A., Mitchell J.F. (1991). Respiratory, acid-base and ionic status of Kemp’s ridley sea turtles (Lepidochelys kempi) subjected to trawling. Comparative Biochemistry and Physiology Part A: Physiology.

[BIBR-23] Stacy N.I., Innis C.J., Hernandez J.A. (2013). Development and evaluation of three mortality prediction indices for cold-stunned Kemp’s ridley sea turtles (Lepidochelys kempii. Conservation Physiology.

[BIBR-24] Turnbull B.S., Smith C.R., Stamper A.M. (2000). Medical implications of hypothermia in threatened loggerhead (Caretta caretta) and endangered Kemp’s ridley (Lepidochelys kempi) and green (Chelonia mydas) sea turtles. Joint Conference of the American Association of Zoo Veterinarians and the International Association of Aquatic Animal Medicine.

[BIBR-25] Wood S.C., Gatz R.N., Glass M.L. (1984). Oxygen transport in the green sea turtle. Journal of Comparative Physiology B.

[BIBR-26] Wyneken J., Mader D.R., Weber E.S., Merigo C., Mader D. (2006). Medical care of sea turtles. Reptile Medicine and Surgery.

[BIBR-27] Zacher L.A., Berg J., Shaw S.P., Kudej R.K. (2010). Association between outcome and changes in plasma lactate concentration during presurgical treatment in dogs with gastric dilatation-volvulus: 64 cases (2002–2008. Journal of the American Veterinary Medical Association.

